# A Systematic Review of Application Progress on Machine Learning-Based Natural Language Processing in Breast Cancer over the Past 5 Years

**DOI:** 10.3390/diagnostics13030537

**Published:** 2023-02-01

**Authors:** Chengtai Li, Ying Weng, Yiming Zhang, Boding Wang

**Affiliations:** 1 School of Computer Science, Faculty of Science and Engineering, University of Nottingham Ningbo China, Ningbo 315100, China; 2 Hwa Mei Hospital, University of Chinese Academy of Sciences, Ningbo 315010, China

**Keywords:** artificial intelligence, breast cancer, machine learning, natural language processing

## Abstract

Artificial intelligence (AI) has been steadily developing in the medical field in the past few years, and AI-based applications have advanced cancer diagnosis. Breast cancer has a massive amount of data in oncology. There has been a high level of research enthusiasm to apply AI techniques to assist in breast cancer diagnosis and improve doctors’ efficiency. However, the wise utilization of tedious breast cancer-related medical care is still challenging. Over the past few years, AI-based NLP applications have been increasingly proposed in breast cancer. In this systematic review, we conduct the review using preferred reporting items for systematic reviews and meta-analyses (PRISMA) and investigate the recent five years of literature in natural language processing (NLP)-based AI applications. This systematic review aims to uncover the recent trends in this area, close the research gap, and help doctors better understand the NLP application pipeline. We first conduct an initial literature search of 202 publications from Scopus, Web of Science, PubMed, Google Scholar, and the Association for Computational Linguistics (ACL) Anthology. Then, we screen the literature based on inclusion and exclusion criteria. Next, we categorize and analyze the advantages and disadvantages of the different machine learning models. We also discuss the current challenges, such as the lack of a public dataset. Furthermore, we suggest some promising future directions, including semi-supervised learning, active learning, and transfer learning.

## 1. Introduction

Every year, more than 500,000 women die from breast cancer worldwide [1]. Although the incidence and mortality rates of breast cancer vary by country [2], it is undeniable that it is extremely harmful to women. The latest data shows that breast cancer is the most frequently diagnosed cancer among women in the United States, excluding nonmelanoma of the skin, and the second leading cause of cancer death in women [3]. The applications of artificial intelligence (AI) in healthcare began in the 1990s and quickly shifted to oncology [4], where breast cancer has a high prevalence and a large amount of data from related studies. As a result, the applications and methods of AI in breast cancer have been steadily developing so far, especially for data-driven machine learning (ML). ML is a branch of AI, and deep learning (DL) is a subfield of ML [5]. In addition, the remaining ML-based methods are referred to as conventional ML-based methods. ML strives to develop computer systems that automatically improve their performance through experience [6]. Learning from experience means that ML models can use feature layers to learn task-specific representations from the raw data. While conventional machine learning requires humans to formulate feature layers, deep learning involves models learning feature layers from data by themselves [7]. The emergence of ML is a revolution at the intersection of computers and medicine. The advanced machine-learning techniques made it possible to design intelligent medical computer applications to assist doctors in making better decisions. In addition, it can enrich the patient-doctor relationship [8].

Natural language processing (NLP) with ML models is a hot topic in breast cancer applications. The NLP studies how computers can process and understand human language, which is an important direction in the field of computing and artificial intelligence. In the field of breast cancer, we are constantly researching ML-based NLP methods to automate some time-consuming manual tasks. For example, doctors can use ML-based NLP to extract or predict medical variables in electronic medical records (EMRs) [9]. Moreover, doctors can utilize ML-based NLP to analyze internet forum posts to better understand patients’ emotions [10]. However, the explosion in the variety of ML-based NLP models in recent years has created confusion and challenges for researchers with medical backgrounds who want to venture into the intersection of medicine and computing. Doctors may not fully understand the ML mechanisms; hence, it is essential to close the research gap between AI researchers and doctors and conduct interdisciplinary research and collaboration [11].

This review attempts to introduce the mainstream conventional ML-based NLP models and DL-based NLP models in the past five years. Our main objective is to help doctors better understand the advantages, disadvantages, and application scenarios of these models through theoretical and experimental analysis. We hope this review can help researchers who do not have a background in NLP get started in this field.

### Contributions

There have been some reviews on the applications of NLP in breast cancer [12,13,14,15,16]. In contrast to other reviews, we focus more on how to integrate theoretical models with concrete applications. The strength of this review is that we tend to show the details of the models in NLP for breast cancers so that the doctors can quickly match the models to their corresponding application scenarios and know why. This advantage can serve as a better guide for new doctors just entering this field. We believe this review allows researchers who are interested in this field to have a more comprehensive understanding of how to choose models when implementing specific applications. This review focuses on three research questions (RQs):

(RQ1): What are the current dominant ML-based models in NLP applications for breast cancer?

(RQ2): What are the challenges in NLP applications to breast cancer?

(RQ3): What are the future model trends in NLP applications for breast cancer?

In conclusion, the main contributions of this paper are:

- We have summarized a comprehensive NLP pipeline for applications in breast cancer;
- We have produced a detailed introduction to the mainstream models of NLP applications in breast cancer;
- We have concluded the challenges of applications of NLP in breast cancer;
- We have presented the future trends of NLP in breast cancer.

## 2. Theoretical Foundation

**Figure 1:**
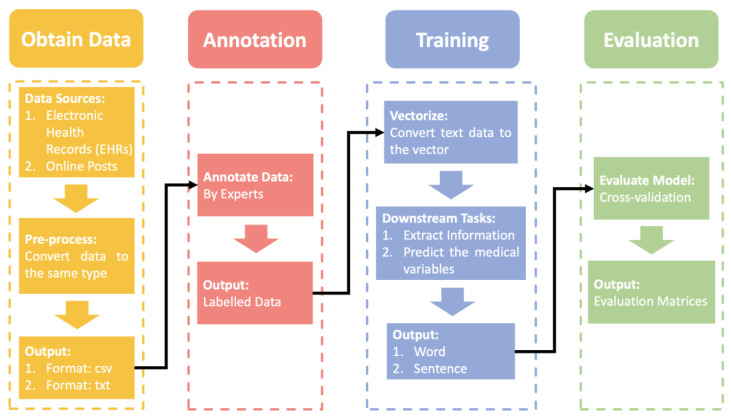
The overview of NLP pipelines in applications of breast cancer is explained.

Prior to introducing the various ML-based NLP models, the NLP pipeline in breast cancer applications should be explained. The following Figure 1 shows an overview of the NLP pipeline.

### NLP Pipeline

First, we need to obtain the raw data, the data sources are usually public or local electronic health records (EHRs) and posts on the web. As raw data, the individuals we collect can vary greatly from one another. We should do pre-processing to unify the text structure. However, before we annotate the data, the data is usually stored in .csv or .txt format. The professional annotation process invites multiple experts to cross-annotate the data several times. Then, we input the labeled data into an ML-based NLP model for training. It should be noted that the data needed for the ML-based NLP model is vectors. Many models can help us convert text into vectors, such as word2vec [17] and bag-of-words [18]. Then, the ML-based NLP model is used to extract the required information or predict medical variables based on different downstream tasks in a step-by-step training process. In the final evaluation phase, we validate the trained model with cross-validation. Cross-validation is more objective and unaffected by imbalances in the validation data set than a one-time validation. After that, evaluation matrices are calculated based on evaluation criteria for quantitative evaluation.

## 3. Materials and Methods

The goal of this review is to introduce the mainstream conventional ML-based NLP models and DL-based NLP models in applications of breast cancer. The focus is therefore on representative and innovative NLP models. We have searched three electronic databases: PubMed, Google Scholar, and the ACL Anthology, for relevant literature between 2018 and 2022. The keywords have been established from three aspects: model-related words (‘**ML**’/‘**DL**’/‘**Machine Learning**’/‘**Deep Learning**’), technique-related words (‘**NLP**’/‘**Natural Language**’), and cancer-related words (‘**Breast Cancer**’/‘**Breast Oncology**’). Table 1 shows the keywords.

**Figure 2:**
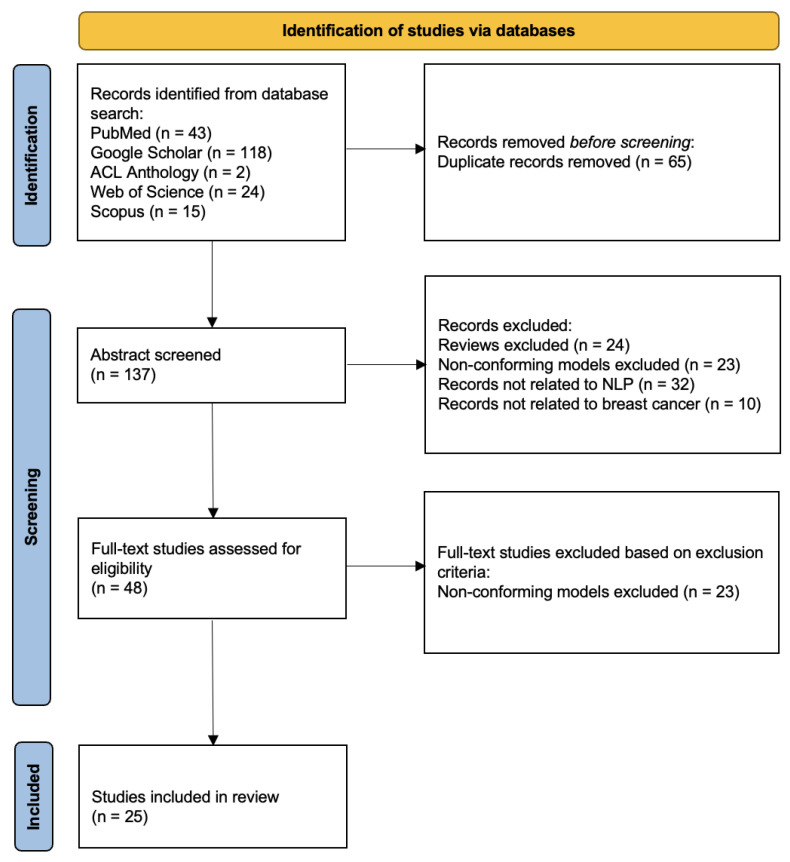
PRISMA diagram for study selection.

In a systematic review, we have followed the process of the preferred reporting items for systematic reviews and meta-analyses (PRISMA). In the above search phase, we have counted a total of 202 papers (43 via PubMed, 118 via Google Scholar, 2 via the Association for Computational Linguistics (ACL) Anthology, 24 via Web of Science, and 15 via Scopus). After removing duplicate records, 137 papers have been chosen for abstract screening. We have selected the papers based on whether the abstract describing the paper is a review, contains conforming models, is related to NLP, or is related to breast cancer. In order to determine the conforming models, we have followed several main points: 1. Papers that simply compare the results of different models have been filtered out; 2. word-embedded models have been filtered out; 3. papers whose purposes are not to develop an NLP breast cancer application have been filtered out; 4. models that are not based on ML have been filtered out. There have been 48 papers that passed the abstract screening. In the full-text screening, we have mainly performed secondary screening of conforming models for papers that do not mention model information in the abstract. Finally, 25 papers have been included in this systemic review. Figure 2 shows the overall process.

## 4. Results

**Figure 3:**
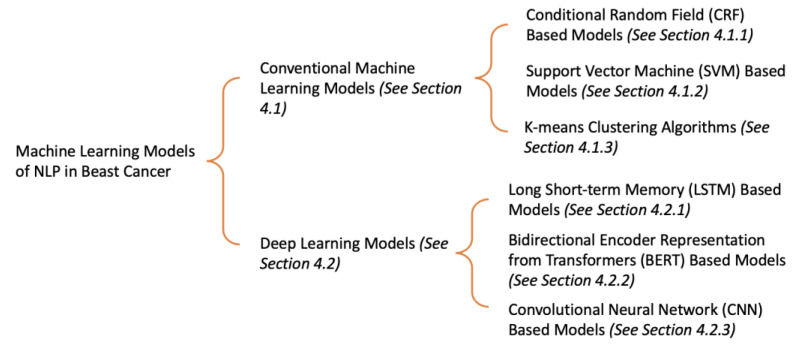
The overview of existing machine learning models of NLP in breast cancer.

In this section, we have mainly divided the machine learning models of NLP in breast cancer into two categories: conventional machine learning models and deep learning models. The conventional machine learning models can be subdivided into conditional random field (CRF) [19] based models, support vector machine (SVM) [20] based models, and K-means clustering algorithms [21]. The deep learning models can be subdivided into long short-term memory (LSTM) [22] based models, bidirectional encoder representation from transformers (BERT) [23] based models, and convolutional neural network (CNN) based models [24]. Figure 3 shows the overview of this section.

In order to enable researchers to better understand the characteristics of each model, we have first introduced the theory of each model, including the advantages and disadvantages, and then presented the relevant breast cancer study for NLP based on the corresponding model.

### 4.1. Conventional Machine Learning Models

In the modern era of high-speed development of deep learning algorithms, conventional machine learning algorithms still have their unique advantages. The conventional machine learning method requires fewer data points and is more interpretable. In addition, many researchers combine traditional machine learning models with deep learning networks to improve the interpretability and robustness of neural networks.

#### 4.1.1. CRF

**Figure 4:**
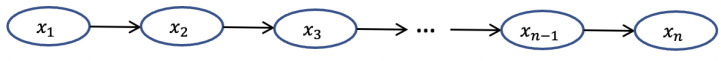
The structure of Markov chain.

In [19], Lafferty et al. presented the CRF model, which is a model to segment and label sequence data. Prior to describing the whole CRF algorithm, we need to introduce the concepts of Markov chains and hidden Markov models (HMM). The Markov assumption is that the state at a specific moment in a stochastic sequence is only related to the state at its previous moment [25]. The Markov chain is a Markov process, which has a discrete state space. The formula of the discrete-time Markov chain is presented below.

(1) $$P\left(x_{n}|x_{1},\;x_{2},x_{3}\ldots x_{n-1}\right)=P(x_{n}|x_{n-1})$$

where x represents the observable state, and $x_{n}$ represents the observable state at *n*th moment, as shown in Figure 4.

**Figure 5:**
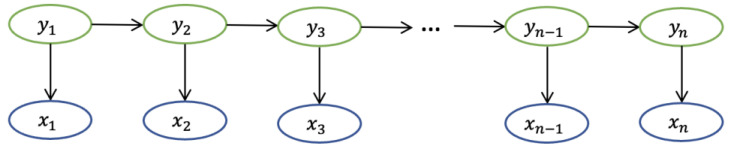
The structure of HMM.

In the Markov chain, each state represents an observable event. However, Markov chains are not sufficient to describe the complex model we wish to discover. For example, we observe not all states but just the observations related to the hidden states. With this limitation, a hidden Markov model (HMM) is designed. HMM has two assumptions: Assumption 1 is that the sequence of hidden states is a Markov chain; Assumption 2 is that each observation depends on the hidden state it corresponds to. The formulas for Assumptions 1 and 2 for the structure of HMM are presented below.

(2) $$P\left(y_{n}|x_{1},\;x_{2},x_{3}\ldots x_{n-1},y_{1},y_{2},y_{3}\ldots y_{n-1}\right)=P(y_{n}|y_{n-1})$$

(3) $$P\left(x_{n}|x_{1},\;x_{2},x_{3}\ldots x_{n-1},x_{n},y_{1},y_{2},y_{3}\ldots y_{n-1}\right)=P(x_{n}|y_{n})$$

where y represents the hidden state, x represents the observation, $y_{n}$ represents the hidden state at *n*th moment, and $x_{n}$ represents the observation at *n*th moment, as shown in Figure 5.

Instead of defining the next state distribution under the current state condition, the CRF model directly calculates the distribution of the entire hidden state sequence given the sequence of observations. The hidden state is related to the entire observation sequence length and contextual information. The CRF model does not have the same strict independence assumption as HMM, thus it can accommodate contextual information. The corresponding formula is presented below.

(4) $$P\left(y_{i}|x_{1},\;x_{2},x_{3}\ldots x_{i-1},y_{1},y_{2},y_{3}\ldots y_{n-1},y_{n}\right)=P(y_{i}|x,y_{i-1},y_{i+1})$$

**Figure 6:**
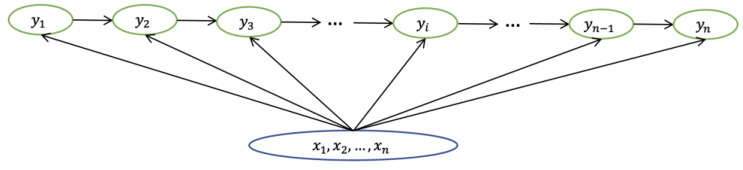
The structure of CRF model.

Where y represents the hidden state, x represents the observation, $y_{i}$ represents the hidden state at *i*th moment, and $x_{i}$ represents the observation at *i*th moment, as shown in Figure 6.

In [9], authors developed an automated system to extract 8 numerical entities using the CRF model from breast pathology reports in Chinese. For instance, the CRF model should identify that “80%” is the ER stain percentage for “The ER percentage value was 80%.” In [26], doctors needed an automated system to assure the quality of patient reports. Pathak et al. applied the CRF model to convert the unstructured text into semi-structured XML text, allowing the report to exist in a more intuitive form. The authors improved the CRF model by transforming a CRF model into a structure containing several CRF models connected by logic while simplifying the overall structure by combining classes. Forsyth et al. [27] built a CRF model capable of extracting patient-reported symptoms. The CRF model is very suitable for this task as the dataset requirement is not high and many features can be captured in the order and connection of words.

The CRF model is suitable for handling serial data such as clinical notes because of its inherent ability to handle contextual relationships. For applications of NLP in breast cancer, one of the application scenarios of CRF is limited by insufficient resources, such as a small data volume or hardware constraints. Whereas, with sufficient resources, the results of the CRF model are worse than those of the deep learning model. The more widespread application scenario is treated as a layer to improve LSTM. We have discussed this application in more detail in Section 4.2.1. We have summarized the advantages and disadvantages of CRF in Table 2.

#### 4.1.2. SVM

The SVM [20] is a classification model whose kernel is to find the hyperplane in the feature space to separate the data and maximize the margin of the samples closest to the hyperplane on both sides. Under the assumption of maximizing margin, the most robust model can be obtained. The example of linearly divisible samples in Figure 4 shows the details of the SVM.

**Figure 7:**
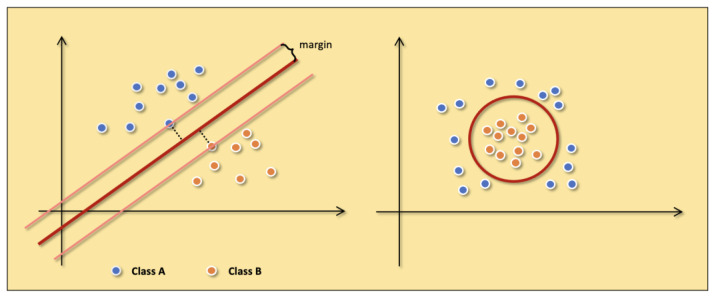
The examples of linearly divisible samples (**the left figure**) and linearly indivisible samples (**the right figure**). Class A (blue points) and Class B (orange points) represent two different samples.

In the ideal case, the data are linearly separable. We can directly follow the basic idea of maximizing the margin to train a linear SVM. However, more real-world data is close to being linearly differentiable. In this situation, the SVM is trained by adding slack variables to maximize the soft margin. The slack variable means we allow the SVM to cause a small number of classification errors when classifying. Moreover, the slack variables not only enable SVM to handle data with near-linear partitions but also alleviate the problem of SVM overfitting. In addition to linearly divisible and approximately linearly divisible data, SVM also deals with linearly indivisible data. The key to handling linearly indivisible data is that we need to map the data to a higher-dimensional space so that the data is linearly divisible. The mapping is done by adding a suitable kernel function to the SVM. The kernel function simplifies the process of mapping the samples from the low-dimensional space to the high-dimensional space and the inner product of the corresponding variables. Figure 7 shows examples of linearly divisible samples and linearly indivisible samples.

Pathak et al. [26] developed the SVM to classify the title and content of patient reports. Ferroni et al. [28] used machine learning for the prognostic classification of breast cancer by developing an SVM-based decision support system. Although this was only an application attempt, the accuracy of the authors’ model in the test set was 86%, which shows that ML algorithms have the potential to obtain prognostic information. In [29], the authors combined SVM with the extra-trees model to identify breast cancers. The extra-trees model was used to filter the features, and SVM was used to diagnose breast cancers. In the experimental section, the authors specifically designed experiments to demonstrate that the use of the extra-trees model allows the selection of breast cancer features that are more favorable to the results. Zexian et al. [30] developed SVM to predict whether patients would have a distant recurrence of breast cancer. In order to improve the accuracy of prediction, the authors made some attempts at feature input. Zexian et al. used MetaMap to filter features from clinical notes and extracted eighteen structured features from the EHR.

The MetaMap is an NLP application for mapping text to the Metathesaurus [31]. There is a lot of meaningful textual information in online forums. In [32], Carrillo-de-Albornoz et al. used SVM based on sequential minimal optimization to automatically classify texts of posts into three categories: experiences, facts, and opinions. Zeng et al. [33] used MetaMap to extract positive features in sentences indicating local recurrence of breast cancer and developed an SVM model to identify local recurrence of breast cancer. The authors compared this model with three baseline models to obtain the best AUC: Using the full MetaMap concept, the filtered MetaMap concept, or the word “package.”

The advantage of SVM is that it can be applied to small sample datasets and is not overly influenced by the dimensionality of the samples. In contrast, the disadvantage of traditional SVM is that it is time-consuming for large-scale datasets because of matrix computation. Table 3 shows the advantages and disadvantages of SVM.

#### 4.1.3. K-means

K-means [21] is an unsupervised machine-learning method that clusters data to distinguish classes. Its algorithm steps are divided into four steps: In the first step, k initial clustering centers are selected. In the second step, for each sample in the dataset, the distance from it to the k cluster centers is calculated and assigned to the class corresponding to the cluster center with the shortest distance. In the third step, the clustering centers are recalculated for each class. In the last step, the second and third steps are repeated until some termination condition, such as a set maximum number of steps or a set minimum difference in cluster center change, is reached.

Huang et al. [34] solved pattern differentiation in breast cancer by using neural networks to unify the terminology of electronic medical records in traditional Chinese medicine (TCM). The authors normalized the data with DeepMedic, which is software used to standardize the TCM terminologies, summarize the TCM pattern, and classify clinical features with K-means. To improve the quality of medical reports, the authors [35] used K-means to learn the structure of medical reports. The results could be used for subsequent structuring of medical reports.

The advantages of K-means are easy to understand, good clustering, and low complexity of the algorithm. While K-means is sensitive to outliers and is not suitable for classes with unbalanced sample sizes or classes that are too discrete, in addition, since samples can only be grouped into one class, K-means is not suitable for multiple-label tasks. We have recorded the advantages and disadvantages of K-means in Table 4.

### 4.2. Deep Learning Models

In the presence of sufficient data, deep learning models have an overwhelming performance advantage over conventional machine learning models in processing NLP-related tasks. In fact, conventional machine learning models are indeed being gradually replaced by deep learning models in some fields that require high precision. However, the results of deep learning are often difficult to interpret because of their “black box” nature.

#### 4.2.1. LSTM

**Figure 8:**
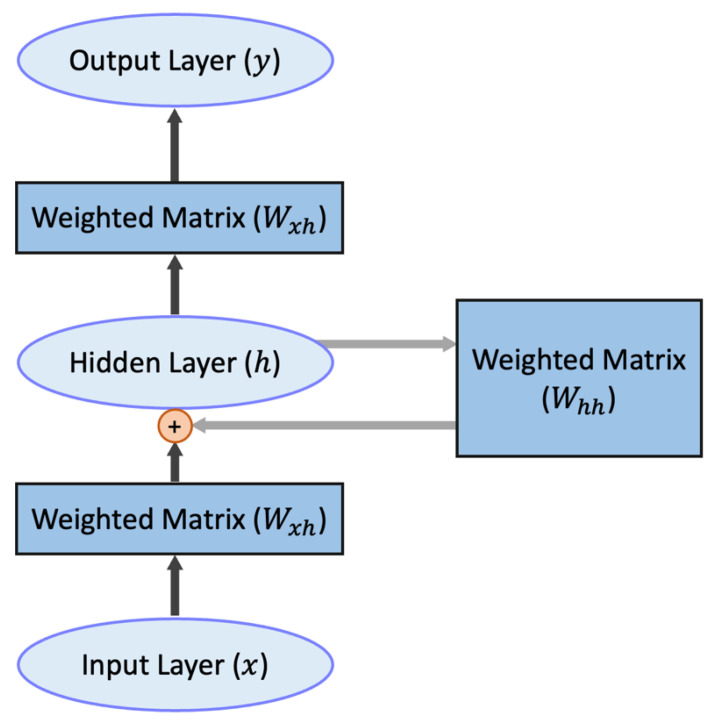
The structure of RNN. The plus sign means the hidden layer *h* is produced by *W_hh_* and *W_xh_* together. The black arrows represent the fully connected part, and the gray arrows represent the recurrent part.

LSTM [22] is an improved version of recurrent neural network (RNN) [36], which is a neural network used to process sequential data. RNN is characterized by its ability to handle contextual relationships well, predicting the next data based on the relationship of the previous sequence data. Figure 8 below shows the structure of the entire network. We can observe that the value of the hidden layer in each training session does not only depend on the input but is also influenced by the hidden value of the previous cycle. RNN is structured in such a way that the previous sequence data affects the later sequence data.

RNN may experience gradient vanishing and gradient explosion during training due to the cyclic iterations of the weight matrix. LSTM consists of a chain of basic units that can solve these problems to some extent with gates and control features. Additionally, a unit consists of an oblivion gate, an input gate, an output gate, and a cell state. The forgetting gate determines how much of the original feature information is discarded. The input gate determines which feature information is updated. The cell state is a cell that stores feature information. The output gate is used to decide which feature information is output. There are some variants of LSTM, such as bi-directional long short-term memory (Bi-LSTM), which not only predicts the current state based on the previous state but also considers the future state.

The chatbots can reduce the burden on healthcare workers while helping to provide advice to many patients. Maktapwong et al. [37] designed a bi-LSTM-based model for text classification to provide a chatbot to breast cancer patients. In [38], the authors used Bi-LSTM-CRF as a pooling layer to identify entities after the output of BERT to enhance model accuracy. Sanyal et al. [39] developed a weakly supervised framework for breast cancer recurrence prediction using LSTM to label the original unlabeled dataset. The experimental results confirmed that training in a semi-supervised framework was better than training with only manually labeled data. Magna et al. [40] developed a recommendation system for the diagnosis of breast cancer based on medical histories. The design of the authors’ experimental section was comprehensive. In order to test the models of word embedding, a comparison of various classical conventional machine learning models and deep learning models, which were based on CNN and LSTM, was presented in terms of performance.

LSTM has sequence dependencies and cannot be processed in parallel. In addition, the gradient problem of RNN is solved to some extent in LSTM, but it is still not enough. It can handle small to medium-sized sequences, while longer sequences will still be tricky. CRF can learn the context of the label, while LSTM alone can only learn the contextual relationship of the feature. On the contrary, the contextual relationship of the label is not learned. The advantages and disadvantages are shown in Table 5.

#### 4.2.2. BERT

**Figure 9:**
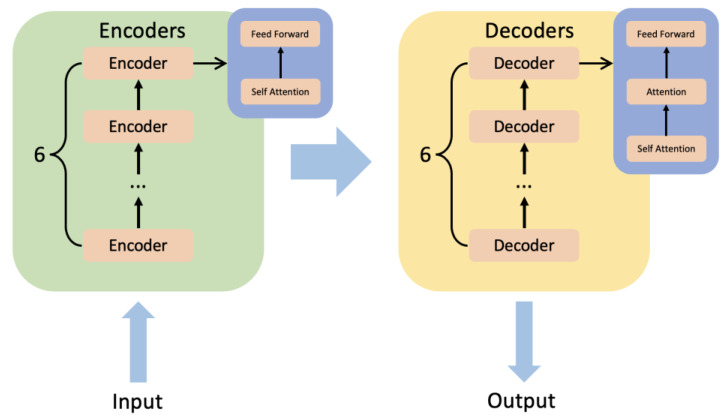
The inner structure of encoders and decoders of transformer.

BERT [23] is a pre-trained fine-tuning model based on transformer [41]. The significance of BERT is that we have satisfied Transformer’s massive parameter training with unlabeled data. The transformer is a model that uses an attention mechanism to improve training speed and accuracy. The structure of the transformer can be simply summarized as encoders and decoders. There are six minor encoders and six minor decoders. Figure 9 shows the inner structure of encoders and decoders. A minor encoder includes a self-attention mechanism and a feed-forward neural network. A minor decoder includes a self-attention mechanism, an attention mechanism, and a feed-forward neural network.

The pre-trained BERT can be divided into two unsupervised learning tasks. In the first task, we set up random masks in the sentences and predict these masks based on the context to obtain the parameters. In order for the model to better understand the relationship between sentences, in the second task, we make the model perform the prediction task for the next sentence. In detail, we put two sentences together and let the model determine whether they are adjacent to each other in the text. The pre-trained BERT can access different fully connected layers when facing different tasks to accomplish task-specific training and prediction. The development of pre-trained models substantially reduces the learning burden for the transformer family models.

The research [38] proposed a BERT-based system, including named entity recognition (NER) and relation recognition, to extract the concepts and attributes. In facing different NLP tasks, the authors proposed two different BERT fine-tuning models: Due to the excellent performance of BI-LSTM-CRF in the NER task, the authors input the semantic vectors extracted by BERT into BI-LSTM-CRF to identify entities. In relational recognition, the authors added a linear classification layer to BERT to predict the labels of candidate pairs. Kuling et al. [42] designed an adjusted BERT model to make the section segmentations. In addition to the original contextual word embeddings, the authors added the auxiliary information vectors to the classifier head to get the significant improvement. The results of section segmentation tasks could be exploited to improve the accuracy of field extraction tasks. Solarte-Pabón [43] applied BERT trained in the lung cancer corpus to extract cancer concepts from the breast cancer dataset. BERT achieved a high F-score when faced with breast cancer data that had never been seen during training. This suggests that the cancer concepts obtained by BERT in the dataset are generalizable and can be used to infer other cancer datasets. In [10], the authors wanted to understand the worries of breast cancer patients from their daily posts. As a result, they applied BERT as an affective classification model and used it to process the texts of breast cancer patients to classify their worries. Zhou et al. [44] pre-trained BERT on a cancer-specific dataset to extract breast cancer phenotypes from clinical texts and discussed the impact of pre-training on a specific corpus on the performance of BERT. The results show that pre-training BERT with a specific corpus can significantly improve its performance. Kumar et al. [45], designed a BERT-based model specifically for Shared Task 8 of SMM4H-2021, which is to classify self-reported breast cancer posts on Twitter. They used BlueBERT [46], which is pre-trained on PubMed’s biomedical corpus. In addition, to enhance robustness, the authors incorporated BlueBERT with gradient-based adversarial training during the training process. In order to build interpretable neural networks, the authors [47] started by embedding semantic trees into BERT and using a capsule network to improve the semantic representation of multiple attention heads. Finally, backpropagation and dynamic routing algorithms allowed the local interpretability of the model. Patient-centered outcomes (PCOs) for breast cancer patients are hard to detect. AI-Garadi [48] designed a classifier based on BERT to identify the self-reports of breast cancer on Twitter. The qualitative analyses of these self-reports made the PCOs feasible to detect.

The advantage of BERT is that it is based on a transformer structure, which is more capable of extracting information compared to LSTM. Moreover, it can extract long-distance relations without the problem of gradient vanishing. The disadvantage is that the pre-training and fine-tuning phases of the task are not exactly matched, which affects its effectiveness. Table 6 shows the advantages and disadvantages of BERT.

#### 4.2.3. CNN

**Figure 10:**
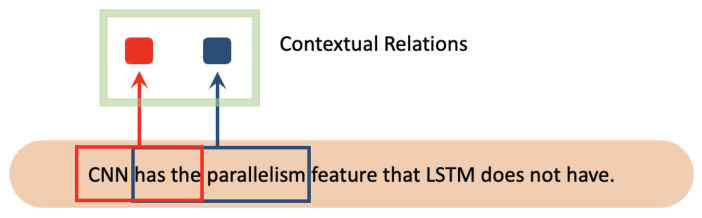
The convolutional processing of CNN. The red color and blue color represent two different contextual relations of corresponding words.

CNN [24] has a parallelism feature that LSTM does not have. It achieves the extraction of contextual relations by performing convolutional calculations via sliding windows for a specific length of text. Figure 10 shows the convolutional processing of CNN.

There is an important concept in CNN called the “receptive field.” The receptive field determines how long CNN can make predictions based on contextual relationships. The parameters that control the receptive field are called the window size and stride used by the convolution. A large window size on CNN results in a large receptive field, and more contextual relations are acquired. However, this weakens the influence of the words closest to the prediction target in position on the prediction results. If the stride of CNN is set large, some contextual relations will be ignored, and the overall computation speed will be fast.

Saib et al. [49] developed a hierarchical CNN system to annotate International Classification of Disease for Oncology (ICD-O) codes for breast cancer pathology reports. The authors built a hierarchical CNN to solve the problem. The parent CNN is a multi-classification CNN that classifies different reports into suitable groups. Whereas the child CNN is a binary classification CNN and a multiclassification CNN to predict the final codes, in [50], authors applied CNN to tweets to filter tweets related to patients’ self-diagnosis. The tweets help healthcare professionals better understand the needs and concerns of their patients. Zhao [51] applied CNN to extract biomarker states from breast cancer patients. In order to solve the problem of inconsistency in linguistics, the authors performed a dual embedding in English and Bulgarian and adjusted the vectors obtained after the embedding so that the vector space they are in is consistent. Wang et al. [52] transformed clinical notes into concept unified identifiers (CUI), which are fed into a variant model of CNN, the knowledge-guided convolutional neural network (K-CNN) [53], to predict the distant recurrence probability of breast cancer patients.

The long-range feature capture capability of CNN is much lower than that of RNN and Transformer, but the comprehensive feature extraction capability of CNN is usually slightly better than the performance of RNN. In addition, due to its high computational efficiency and fast training speed, we can choose CNN if, in the application background, there is a need to get experimental results quickly. Table 7 shows the advantages and disadvantages of CNN.

## 5. Discussion

In this section, we provide answers to three questions from Introduction. Based on the findings and analysis of the review, the answer to RQ1 is addressed in Section 5.1. Moreover, we answer RQ2 and RQ3 in Section 5.3 and Section 5.4, respectively.

### 5.1. Models over the Years

We have counted the model trends used in the publications covered in this review over a one-year span. Figure 8 shows the results.

**Figure 11:**
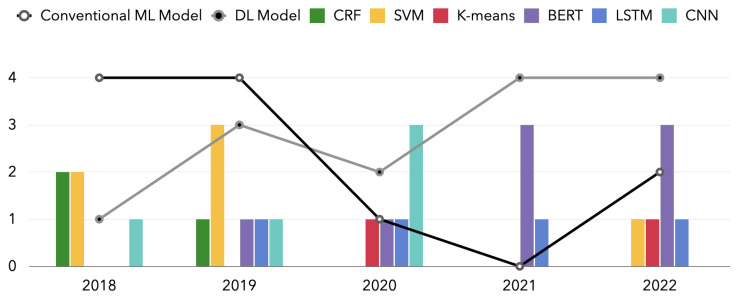
The number of NLP models over years: a comparison of conventional ML model and DL model.

Moreover, from this review, we can also clearly identify the ML-based NLP algorithm paradigm. In Figure 11, we use the collapsed lines to represent the two broad categories of deep learning and traditional machine learning models. For each specific model, we use bar charts for statistics. According to the review results, most studies applied conventional ML models such as CRF and SVM, which were still widely applied in 2018 and 2019. In addition, we find that the use of deep learning models led by BERT has been increasing in the past five years. In contrast, conventional machine learning models, including CRF and SVM, are gradually being replaced by deep learning models. Such a trend is in line with the evolution of machine learning algorithms. Furthermore, in a field such as medicine, where high-precision results are required, a gradual focus on deep learning is to be expected. High-performance models such as BERT with many parameters will be increasingly applied in breast cancer, even completely replacing past models for some tasks. However, we do not think there is any more development space for conventional machine learning models. With limited hardware conditions and data sets, traditional machine learning is still a suitable option. In addition, conventional ML can be combined with DL to give researchers new scope for exploration. In some specific scenarios, the conventional ML model can be used as the result classification behind the DL model, which is only used to extract features. The review results also indicate the trend of a paradigm shift in which most commonly used ML-based NLP algorithms have changed from conventional ML algorithms to CNN, RNN models, and transformer-based models such as BERT.

Additionally, regardless of the type, NLP models in the field of breast cancer are valuable for application. Researchers are often motivated to design NLP models for a specific medical problem, for how patients behave in society, or to design a system. Most of these models are then validated with some careful multi-institutional data for generalizability before they can become practical products in life. Although somewhat limited by the data, the accuracy, or f-score, of these NLP models in the breast cancer field basically achieved a desirable value.

### 5.2. Dataset Information

Table 8 provides statistics on the size of the dataset used for the articles covered in this review. The size of the data set for the online forum is large. This corroborates the high number of people with breast cancer, and the amount of data on breast cancer should be large. However, the size of some of these datasets is much smaller than other types of NLP datasets, such as general language understanding or sentiment analysis. This is because the medical text is inherently highly specialized and technical. In the human annotation segment, researchers have a deep understanding of the kind of highly specialized datasets that are required. A significant increase in human costs is involved. Thus, this leads to the small size of most of the medical datasets. During the process of collecting the data set and the study, researchers need to consider patient privacy. Most of the datasets are private.

In Table 9 below, we have provided links to publicly available datasets covered by studies.

Three of the publicly accessible datasets we have listed are websites for researchers to find relevant posts: Life Palette, Twitter, and MedHelp. The eDiseases Dataset contains annotated sentences about breast cancer from MedHelp. The Breast Cancer Coimbra dataset is based on 10 quantitative predictors and a binary dependent variable indicating whether there is breast cancer. The MIMIC-III contains information about patients admitted to the intensive care unit. The I2B2 study dataset is composed of fully de-identified notes. Oncoshare is a breast cancer dataset that was developed by Stanford Health Care. The entire Oncoshare is not available for use. However, the de-identified subset is available on request [39]. The EMRs for breast cancer from the China Medical University Hospital (CMUH) database are available on request [34].

Furthermore, by excluding the publicly available text data from the forum, the clinical public datasets for breast cancer are inadequate. In most cases, we still need to rely on the collection of private datasets if we want to develop NLP models with practical implications.

### 5.3. Challenges

Watanabe et al. [10] proposed that the amount of data in their dataset is insufficient for each label. This limitation affected the accuracy of their model. Moreover, Mektapwong et al. [37] mentioned that if there were more learning data, the answers of their chatbot may be like human interaction. Huang et al. [34] raised another issue of data in their research. In the case of all data coming from the same medical center, there is a potential for selective bias in the data. In addition, Tang et al. [9] stated in their future work that they will collect data from another institution to test whether the model can be generalized to different institutions. We considered that this also indicated a possible selective bias in the data coming from the same institution. Zhao [51] discovered that the training data contained some errors. Part of these errors were caused by the experimenter’s annotation, while others came from the medical records or registers [62].

The challenges for the development of machine learning-based NLP for breast cancer applications are mainly around datasets. On the one hand, according to Section 5.2, we can conclude that there is a lack of public datasets for NLP studies in breast cancer. This means that we need higher research costs to obtain private datasets for scientific research. The private datasets are limited by the size of the study, which creates the problem of possible selectivity bias. The model developed for one private dataset may not have general applicability. It is likely that the model cannot be used on other private datasets of the same type because of the data format and annotation. In addition, the lack of public datasets will make the experimental results less available and mean that they cannot be compared with other experimental results of the same type. On the other hand, even if we have access to private datasets, the records in the private dataset may contain some errors. The quality and quantity of private datasets are also difficult to guarantee. Unbalanced and small datasets will limit the performance of the model. Especially in deep learning networks with complex structures, small-scale datasets have small feature sets and are prone to overfitting. What we need to recognize is that models are not our core competency in the application of AI in the medical-related field represented by breast cancer. There are too many suitable models for us to choose from and improve now. What we often lack is an adequate dataset to train and test the models. The core challenge is to find data sets with the required quantity and quality of data.

### 5.4. Future Directions

Due to privacy, policy, and cost, we have difficulty solving the problem of a few public datasets. However, we can increase the utilization of private data and solve the challenges of datasets to some extent. Three solutions are involved here: semi-supervised learning, active learning, and transfer learning.

#### 5.4.1. Semi-Supervised Learning

Semi-supervised learning is a branch of machine learning that aims to combine supervised learning and unsupervised learning [63]. Semi-supervised learning tries to improve the performance of supervised learning by using relevant information from unsupervised learning. This means that semi-supervised learning can be trained using unlabeled data; for example, we can add unlabeled data points to a classification problem to help the classification process. In the prospect of NLP applications for breast cancer, we can improve the model’s accuracy with the help of semi-supervised learning and a lot of raw data without adding any cost to the performance.

#### 5.4.2. Active Learning

The key assumption of active learning is that the model chooses the data to learn from [64]. The goal of active learning is to achieve the best possible performance of the model using as few high-quality sample annotations as possible. The significance of this is that the cost of labeling is reduced. Typically, in a regular pairwise task, we would randomly select from the samples to provide the samples to be labeled for manual labeling. However, active learning uses machine learning to select suitable candidate datasets for people to label and iterate on to get a better performing model. This is where active learning differs from semi-supervised learning.

#### 5.4.3. Transfer Learning

The kernel of transfer learning is to improve the model of a domain by transferring information from related domains [65]. The essence of transfer learning is to adapt an existing model to a new dataset. Transfer learning reduces the cost of building a model from scratch and can significantly minimize the need for training data and training time in the target domain. In the medical domain, we can use large datasets from other domains to train the model. Then a small number of datasets are used to transfer the model to the corresponding medical domain.

## 6. Conclusions

The NLP models based on machine learning can assist doctors with tedious medical texts in a high-performance manner, helping them to conduct more research in breast cancer. In addition, to the best of our knowledge, few relevant reviews have been able to discuss in detail the machine learning models involved in the study. To fill this gap over the past five years, we have conducted a literature review of PubMed, ACL Anthology, Google Scholar, Web of Science, and Scopus between 2018 and 2022, resulting in the inclusion of 25 papers. There have already been reviews that summarized the NLP models in breast cancer before 2018. Our review can be a supplement to 2018–2022. We have analyzed these articles and classified them according to the models they are based on: conventional ML-based models and DL-based models. We have found that DL-based models have been increasingly used in the past five years, which is in line with the general trend of machine learning model development. In addition, we have analyzed the dataset to identify the current challenge. The challenge is that there are inadequate publicly available datasets, and the private datasets have some quantitative and qualitative limitations. Based on these challenges, we propose some future research directions, such as semi-supervised learning, transfer learning, and active learning. These directions all focus on how to train models with a small number of labeled datasets that can be investigated to address these challenges. We believe that this review will help medical professionals better understand the current AI field and provide the necessary support for future researchers to design NLP applications in breast cancer.

## Figures and Tables

**Table 1:** Keywords for research related to ML-based NLP in breast cancer applications.

Model-Related Words	Technique-Related Words	Cancer-Related Words
AI/ML/DL/Machine Learning/Deep Learning/Artificial Intelligence	NLP/Natural Language Processing	Breast Cancer/Breast Oncology

**Table 2:** The advantages and disadvantages of CRF.

Advantages	Disadvantages
Suitable for handling serial data	Low performance ceiling
Cheap training environment	
Can be a component for improving LSTM	

**Table 3:** The advantages and disadvantages of SVM.

Advantages	Disadvantages
Can be applied in small sample datasets	Time consuming in large scale datasets
Not overly influenced by dimensionality of samples	

**Table 4:** The advantages and disadvantages of K-means.

Advantages	Disadvantages
Easy to understand	Sensitive to outliers
Good clustering	Not suitable for classes with unbalanced sample classes
Low complexity	Not suitable for overly discrete classes
	Not suitable for multiple-label tasks

**Table 5:** The advantages and disadvantages of LSTM.

Advantages	Disadvantages
Suitable for handling serial data	Cannot be processed in parallel
	The training process still contains gradient problems.
	Can only learn the contextual relationship of feature

**Table 6:** The advantages and disadvantages of BERT.

Advantages	Disadvantages
Can extract contextual relationships of long sequences	The pre-trainig and fine-tuning phases of task are not exactly matched

**Table 7:** The advantages and disadvantages of CNN.

Advantages	Disadvantages
Computational efficiency and fast training speed	Not good at long distance capture features

**Table 8:** The information of publications’ datasets. Private represents the dataset is not available. Public represents the dataset is available.

Reference	Year	Type	Size
[42]	2022	Private	Pre-training: 155,000 breast radiology report, Fine-tuning: 900 breast radiology report
[43]	2022	Private	Lung cancer corpus: 14,000 sentences Breast cancer corpus: 200 sentences
[29]	2022	Public	116 subjects
[10]	2022	Public	2272 breast cancer posts
[37]	2022	Private	1139 messages
[35]	2022	Private	14,105 sentences
[44]	2021	Private	Pre-training: 4,543,184 clinical notes and 1,278,805 pathology reports, Fine-tuning: 9685 sentences
[45]	2021	Public	5019 tweets
[47]	2021	Private	2857 mammography data
[39]	2021	Public	892,550 clinical notes
[34]	2020	Public	2738 records
[40]	2020	Public and Private	Private: 49,475 records, Pulic: 61,464 records
[48]	2020	Public	5019 tweets
[52]	2020	Private	6447 patients
[32]	2019	Public	479 posts
[28]	2019	Private	454 patients
[26]	2019	Private	For heading and content identification: 180 reports, For automatic structuring: 108 reports
[38]	2019	Private	8473 sentences
[51]	2019	Private	2246 records
[9]	2018	Private	2026 breast pathology reports
[27]	2018	Private	10,000 sentences
[49]	2018	Private	2201 breast cancer pathology reports
[33]	2018	Private	701 subjects
[50]	2018	Public	1000 tweets
[30]	2018	Private	1995 subjects

**Table 9:** The links to publicly available datasets.

Dataset	Link	Reference
Breast Cancer Coimbra Dataset	https://archive.ics.uci.edu/ml/datasets/Breast+Cancer+Coimbra (accessed on 15 January 2023)	[54]
Blog Articles on Life Palette	https://lifepalette.jp	[55]
Tweets from Twitter	https://twitter.com/iamfireprhoof/status/1570039829378875392 (an example of tweets) (accessed on 15 January 2023)	[56]
Text from MedHelp	http://www.medhelp.org (accessed on 15 January 2023)	[57]
Oncoshare Breast Cancer Database	https://med.stanford.edu/oncoshare.html (accessed on 15 January 2023)	[58]
I2B2 NLP Research Database	https://www.i2b2.org/NLP/DataSets/Main.php (accessed on 15 January 2023)	[59]
MIMIC-III Critical Care Database	https://github.com/MIT-LCP/mimic-code (accessed on 15 January 2023)	[60]
eDiseases Dataset	https://zenodo.org/record/1479354#.Y8P4kexBy3I (accessed on 15 January 2023)	[61]
China Medical University Hospital (CMUH) database	/	[34]

## Data Availability

Not applicable.
